# Single‐Step Production of Mannitol From Yacon Fructo‐Oligosaccharides Using Fructophilic Lactic Acid Bacteria

**DOI:** 10.1002/mbo3.70387

**Published:** 2026-09-22

**Authors:** Arun S. Rajkumar, John Leech, Olivia McAuliffe

**Affiliations:** ^1^ Teagasc Food Research Centre, Moorepark Fermoy Cork Ireland; ^2^ School of Biological Sciences Queens University Belfast Northern Ireland; ^3^ Teagasc Climate Centre, Moorepark Fermoy Cork Ireland

## Abstract

Mannitol is a naturally occurring sugar alcohol with several applications in the food and pharmaceutical industries, with special interest as a low‐calorie sweetener. While mannitol can be synthesized chemically, synthesis by bacterial fermentation is an attractive means of production due to its low cost, mild (near‐ambient) conditions for synthesis and high yield. Bacterial production also allows the possibility of using low‐cost, second‐generation feedstocks to produce mannitol in a sustainable manner. In this work, we describe the feasibility of producing mannitol from yacon (*Smallanthus sonchifolius*) tuber using *Leuconostoc mesenteroides* and *Fructobacillus fructophilus* previously isolated from floral sources. The tuber contains over 50% fructooligosaccharides (FOS) which was found to be bioavailable for mannitol production without prior hydrolysis or saccharifying pretreatments. Initial screening of several fructose‐tolerant lactic acid bacteria (LAB) led to the selection of the most robust mannitol‐producing strains for optimizing culture conditions and scale‐up. We ultimately demonstrated the successful production of up to 7 g/L mannitol using *L. mesenteroides* with 2% yacon root powder in 200 mL bioreactors, achieving a yield greater than 0.6 g mannitol/g FOS with no significant decrease in titers as the scale of culture is increased. Our study demonstrates that yacon powder is a suitable and cheap substrate for the sustainable and economical production of an important sugar alcohol by biotransformation. Finally, we discuss future approaches to increase the scale and purity of production.

## Graphical abstract

Mannitol is a sweetener with a low glycaemic index and calorific value that can be produced efficiently by bacterial transformation. We demonstrate its production by *Leuconostoc mesenteroides* and *Fructobacillus fructophilus* from a novel substrate, fructo‐oligosaccharides from the tuber yacon in a single‐step process that is amenable to scale‐up.

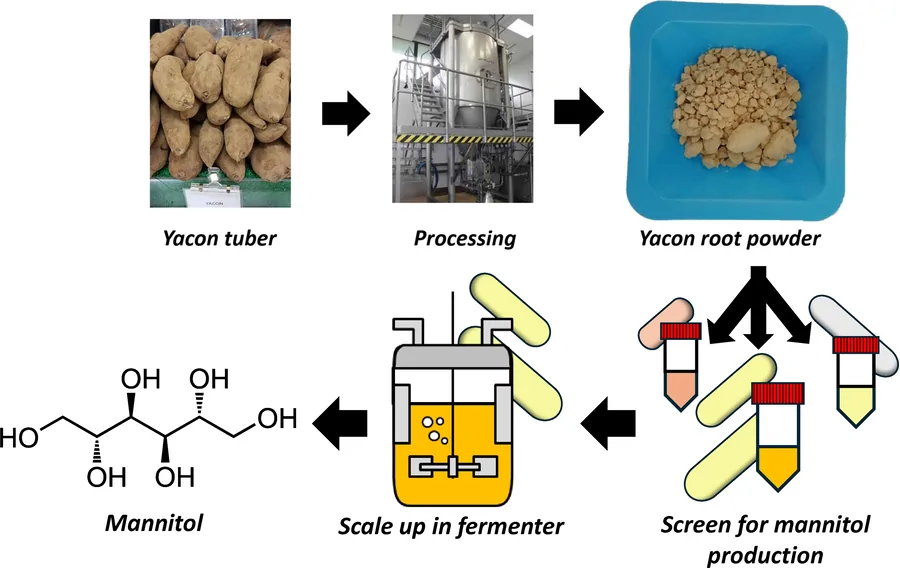

## 1 Introduction

Diseases of affluence such as diabetes, cardiovascular disease and obesity are often associated with the increased consumption of processed foods rich in sugar. There is therefore an interest to replace sugars in such foods with sweeteners with fewer health risks. One such sweetener is mannitol, a six‐carbon polyol with multiple commercial applications beyond the food sector. As with sugar alcohols like sorbitol and xylitol, mannitol is an EU‐approved sweetener (E421) with a low glycaemic index and a low calorific value (Grembecka 2015), making it an attractive choice in low‐calorie or diabetic‐approved sweeteners and sweet foods. The global mannitol market value was estimated to be $451 million in 2024, with growth of 5% expected over the next 5 years (Grandview 2024). At present, it is produced industrially by the chemical reduction of invert sugar at high pressures and temperature (Soetaert et al. (1999). However, this process can create a large amount of sorbitol (up to 75%) as a side product, increasing the complexity of downstream processing (Deis and Kearsley 2012; Martínez‐Miranda et al. 2022).

An alternative to this process is the microbial production of mannitol by fermentation (Yun and Kim 1998). Mannitol is a naturally‐occurring metabolite in several microbes used for energy storage, protection, osmotic stress, or redox balancing. It is typically synthesized from fructose by a single enzymatic reaction. As a potential route for industrial mannitol production, microbial biosynthesis has a number of advantages: production at atmospheric pressure and mild temperatures (30°C–37°C), reducing the need for expensive catalysts, simpler recovery of mannitol from fermentation broth and the use of cheap feedstocks from food and agricultural wastes in place of pure sugars (Martínez‐Miranda et al. 2022). The latter is especially relevant if the microbial production of mannitol is to be sustainable and economically viable; a low‐cost feedstock would drive down production costs and permit a competitive market price. Besides using fructose or sucrose directly, a number of suitable plant‐derived feedstocks have been tested so far to produce mannitol by fermentation. The main requirements of such feedstocks are the presence of fructose, or a more complex carbohydrate that contains fructose as a component (Fontes et al. 2009; Carvalheiro et al. 2011; Ortiz et al. 2012; Rice et al. 2020). However, in the case of more complex feedstocks, oligosaccharides are typically saccharified or otherwise hydrolyzed independent of the actual fermentation step (Saha 2006b; Cao et al. 2018), and the consolidated bioprocessing of feedstock to mannitol would be a more streamlined process for scale‐up. Several microbes have the potential to simultaneously saccharify fructans (polymers of fructose), and convert them to mannitol. The best‐known fructan is inulin, a linear polymer of at least 10 fructose monomers used as a storage compound in several tubers and roots. We propose that the use of low‐degree‐of‐polymerization substrates such as yacon FOS could enable more rapid microbial utilization, thereby reducing fermentation time and eliminating the need for separate enzymatic saccharification, with associated process and cost benefits (Saha 2006b).

In this study, we demonstrate the use of heterofermentative fructophilic lactic acid bacteria (FLAB), isolated from various flower species, to convert yacon root powder (YRP) to mannitol. Yacon (*Smallanthus sonchifolius*) is a tuber from the Andean regions of South America, and several of its cultivars have a high fructooligosaccharide (FOS) and antioxidant content (Choquechambi et al. 2019). Yacon has been cultivated successfully in Ireland for its potential prebiotic effects and low glycaemic index (Beotanics 2019), with several varieties performing well under local conditions, highlighting its potential as a high‑value cultivation for temperate climates such as in Ireland. The FLAB, mostly *Leuconostoc* and *Fructobacillus* species, are able to directly break down the FOS to fructose and convert it to mannitol. When used alongside glucose in the medium, the glucose supports the growth and general metabolism, while the fructose derived from FOS is used exclusively for mannitol production. We demonstrate for the first time that FLAB strains can convert YRP to mannitol at different scales and substrate loading. We have screened 10 FLAB strains for their ability to robustly produce mannitol. Following this screen, we optimize growth and production parameters for a flower isolate of *Leuconostoc mesenteroides*. and successfully scale up its production ten‐fold with no significant loss in titer. These findings establish yacon root powder as an attractive feedstock for the sustainable production of mannitol via bacterial fermentation.

## 2 Materials and Methods

### 2.1 Media and Components

Yacon root powder (YRP), prepared by spray‐drying yacon syrup, was obtained from Beotanics (Stonyford, Co. Kilkenny, Ireland). FOS and free sugar content were analyzed by Eurofins Food Testing (Dublin, Ireland) using a method derived from AOAC 999.03. All bacteria were revived from glycerol stocks on Man‐Rogosa‐Sharpe agar (MRS; Fisher Scientific Ireland; Dublin, Ireland). Subsequently, they were grown in MRS broth supplemented with 1% fructose (Fisher Scientific, FMRS). For the production of mannitol, bacteria were grown in FMRS or MRS supplemented with YRP (YMRS). YMRS broth was prepared by dissolving 1%–2% YRP in reconstituted MRS broth, centrifuging the solution at 5000 rpm/4686 g for 10 min to sediment any insoluble components of YRP and sterilizing the supernatant by autoclaving or filtration. In the case of YMRS used for benchtop fermentations, the medium was made up from individual components (all obtained from Fisher Scientific or Avantor Ireland), but with Tween 80 (polysorbate) omitted, centrifuged and the supernatant autoclaved and used for fermentations. Tween 80 was omitted as it caused a precipitation of YRP in the medium after autoclaving and interfered with cell density measurements. The final pH of YMRS was between 6.1 and 6.3 and was not adjusted prior to autoclaving. Chemicals used for HPLC analysis of metabolites were purchased from Fisher Scientific or Merck Life Science (Arklow, Ireland), while HPAEC standards were purchased either from Merck, Neogen (Bray, Ireland) or Biosynth (Compton, UK).

### 2.2 Microbial Strains and Culture Conditions

Strains of interest were revived from frozen glycerol stocks consisting of MRS medium with 30% glycerol onto MRS agar and incubated at 30°C. Unless specified, all bacteria came from the DPC Culture Collection at Teagasc (Moorepark, Fermoy, IE). Other strains were obtained from the DSMZ German Collection of Microorganisms and Cell Cultures GmbH (Braunschweig, Germany). Colonies from MRS agar plates were grown overnight (usually 20–24 h) as precultures in FMRS at 30°C. These were diluted to 1% (equivalent to a starting OD of 0.03–0.06) in a 15 mL culture tube with 7.5 mL fresh MRS containing 1% fructose, 1%–2% YRP or no supplemental carbohydrate for the evaluation of mannitol production at 30°C with 150 rpm shaking. For mannitol and metabolite analysis, 0.5 mL of culture was taken at different time points, centrifuged for 2 min at 13,000 rpm/18,000 *g* in a microcentrifuge (Eppendorf 5424, Fisher Scientific, IE) and the supernatant stored for analysis. In the course of evaluating mannitol production capability on YRP, we varied the culture temperature, agitation, and YRP loading and investigated their effects on mannitol production.

### 2.3 Scale‐Up Fermentations

Scale‐up fermentations at 30°C and 37°C were conducted in 250 mL benchtop Minibio reactors (Gettinge, Gothenburg, Sweden) at a volume of 200 mL. Temperature was set at 30 or 37°C and with constant agitation at 200 rpm. No aeration was provided and the medium pH was not adjusted as fermentation proceeded. Parameter control and monitoring were performed by myControl controllers through the Lucullus Process Information Management System software suite (Securecell, Urdorf, Switzerland). The medium used for the fermentations was 2% YMRS without Tween 80. The medium was clarified by centrifugation and sterilized in the reactors by autoclaving. Each FLAB strain of interest was grown overnight in FMRS at 30°C was inoculated at a 1% dilution to each fermenter, typically resulting in an OD of 0.05, and the fermentation was run for 48 h. 2 mL samples were taken at 0, 4, 8, 20, 24, 28, 32, 44, and 48 h using the reactors' sampling ports, centrifuged for 2 min at 10,000 rpm/11,000 *g* in a microcentrifuge and stored at −20°C for analysis. Optical density measurements were made on a Jenway SP‐200 spectrophotometer (Cole‐Palmer, St.Neots, UK) at 600 nm, using water as a blank. To convert OD to cell dry weight, a conversion factor of 0.375 mg/OD was used based on prior measurements of cell dry weight and OD of 5 mL cultures of *L. mesenteroides* grown on YMRS.

### 2.4 Analysis of Mannitol and Sugar Consumption by HPLC

Mannitol, glucose and fructose levels were quantified by HPLC using an Aminex HPX‐87P column (Bio‐Rad/Accuscience; Naas, Ireland) equipped on a Waters 2695 HPLC machine and a Waters 2414 refractive index detector. MilliQ water was used as solvent at flow rate of 0.6 mL/min, and the column was heated to 60°C. Ethanol, lactic and acetic acid concentrations were in turn quantified using an Aminex HPX‐87H column heated to 60°C, using 5 mN H_2_SO_4_ as a solvent. In both cases, the refractive index detector was warmed to 35°C and a sample injection volume of 30 µL was used for analysis. Samples were typically diluted 100‐fold. Concentrations of all analytes were estimated from calibration curves of external standards within the range of 20–200 mg/L. When required, mannitol yield from FOS was calculated as the ratio of mannitol titer (in g/L) to the amount of FOS present in the FMRS at the start of fermentation (in g/L). Differences in mannitol titers between strains or experimental conditions were tested for statistical significance using a paired two‐tailed Student's *t‐*test assuming unequal variance and normal distributions on sets containing at least 3 replicates.

### 2.5 Analysis of Yacon Root Powder FOS by HPAEC‐PAD

Fructo‐oligosaccharides were quantified by high performance anion exchange chromatography with pulsed amperometric detection (HPAEC‐PAD) on an ICS6000 system equipped with an electrochemical detector (Dionex, Sunnyvale, CA, USA). Samples were diluted 100‐fold and 10 µL of the diluted samples were injected onto a CarboPac PA100 (2 × 250 mm) column. Eluent A was 100 mM NaOH and eluent B was 100 mM NaOH + 500 mM sodium acetate. Elution was carried out at a flowrate of 0.25 mL/min with the following gradient: *t* = 0–3 min 95% Eluent A; *t* = 3–13 min 88% Eluent A; *t* = 13–17 min 84% Eluent A; *t* = 17–20 min 84% Eluent A; *t* = 20–22 min 95% Eluent A; 22–35 min equilibrated at 95% Eluent A. Both detector and column compartments were operated at 32°C. The quantification of fructo‐oligosaccharides in the samples was performed via a calibration curve obtained with 25 and 50 µg/mL concentrations of each standard.

## 3 Results and Discussion

### 3.1 Composition of Yacon Root Powder

Prior to processing, powdered yacon contained 67% (w/w) FOS (Beotanics, personal communication). Yacon root powder in turn (YRP) contains 54.8% (w/w) FOS, compared to 28% FOS present in the syrup used to produce it. The powder also contained 19.7% fructose, 6.2% glucose, 5.25% sucrose and < 0.3% galactose, lactose and maltose combined, totaling 31.1% free sugars. Independent HPLC measurement of free sugars in YRP solutions returned similar results (35% glucose/fructose/sucrose). The free sugar composition in the powder remained constant when stored in a cold room at 4°C for over a year. Besides increasing shelf life, freeze‐drying the syrup to YRP facilitates handling and precise formulations. Autoclaving YRP solutions did not significantly change the percentage of free sugars as measured by HPLC. FOS from yacon has previously been reported to have a degree of polymerization (DP) of not more than 10 (Goto et al. 1995). Based on information from the manufacturer, 77% of FOSes in the yacon preparation from Beotanics had a DP < 10 and over half had a *DP* between 3 and 6 (Beotanics, private communication). HPAEC analysis of the YRP revealed that most FOS had a DP between 4 and 6. This is reflected by a YRP solubility of up to 10% (w/w) in water. As mentioned above, a small portion of YRP was insoluble and was routinely removed prior to autoclaving as it led to the formation of flocculating precipitates in MRS. Tween 80 in the MRS formulation was identified to be responsible for this and as a result, YMRS without Tween was used for scale‐up experiments. No apparent increase in cell clumping for cultures grown in this medium was observed. However, autoclaved or filter‐sterilised YMRS at concentrations of 2% YRP and above were found to create precipitate over time if not clarified, although this was not associated with a decrease in sugar concentration.

### 3.2 Mannitol Production From Yacon Root Powder by Fructophilic Lactic Acid Bacteria

Having previously identified a number of fructophilic lactic acid bacteria (FLAB) belonging to *Leuconostoc mesenteroides* and *Frutobacillus fructosus* able to convert fructose to mannitol (Behare et al. 2020a), a selection of these strains were screened for their ability to produce mannitol from YRP. The FLAB in question were previously shown to tolerate up to 40% fructose, to possess the mannitol dehydrogenase gene, and to positively modify the sensory and gelation properties of milk. Related *Leuconostoc* and *Fructobacillus* species have been used to produce mannitol in other work (Ruiz Rodríguez et al. 2017; Zhang et al. 2017), but this is the first time FLAB from these sources have been tested on an unhydrolysed FOS‐containing substrate. Unhydrolyzed FOSes or inulin have been typically used for the production of lactic acid or as a substrate for value‐added fermentation of foods (Choi et al. 2012; Petrova et al. 2015), and inulin‐rich substrates such as Jerusalem artichoke, chicory or dahlia are often hydrolyzed prior to fermentation to mannitol (Saha 2006b).

**Figure 1:**
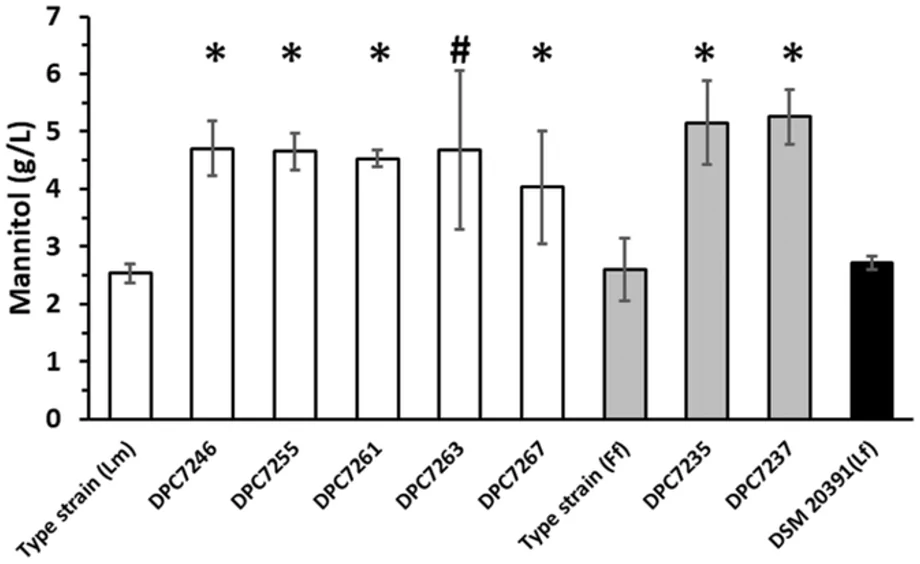
Production of mannitol from yacon root powder by fructophilic lactic acid bacteria. The strains were cultured in MRS broth containing 1% yacon root powder (1% YMRS) and mannitol measured in the medium after 20 h culture as described in the Methods. The bars are colored by species: white are Leuconostoc mesenteroides (Lm), gray are Fructobacillus fructosus (Ff) and black is Limisolactobacillus fermentum (Lf). Full details of the strains are provided in Table 1. Data are plotted as the mean ± s.d. of at least three (3) replicates as error bars. Mannitol titers for each strain significantly higher than that produced by the respective type strains are labeled with an asterisk for *p* < 0.005 or a hash for *p* < 0.05.

This study aimed to employ intact fructooligosaccharides in a single‐step biotransformation to mannitol. As the FOS in YRP are less complex than inulin, we evaluated whether the endogenous invertases or other inulinases expressed by the strains could hydrolyze FOS to fructose for mannitol production. The strains selected for mannitol production screening are listed in Table 1. Genome analysis of *L. mesenteroides* DPC7261 (assembly number GCA_014854895.1) and *F. fructosus* DPC 7238 (assembly number GCF_014489725.1) (Behare et al. 2020b) identified two genes in the *L. mesenteroides* genome that code for glycoside hydrolases with the GH32 domain, which are likely to be inulinases or invertases based on the CAZy database at (https://www.cazy.org) (Drula et al. 2022). These data suggest that the fructophilic *L. mesenteroides* strains are likely to degrade the FOS in YRP. Based on these considerations, a greater number of *L. mesenteroides* strains were selected for screening than *F. fructosus* strains. All FLAB strains selected for screening produced 4–5 g/L on mannitol when grown in 15 mL MRS containing 1% YRP (1% YMRS) at 30°C, whereas the type strains for the two species produced less than 3 g/L on 1% YMRS (Figure 1). The difference in titer between type strain and DPC strains was more prominent in *F. fructosus*, with the DPC strains producing twice as much as the type strain (5.2 ± 0.48 g/L vs. 2.6 ± 0.54 g/L). Another LAB reported to produce mannitol from fructose or saccharified fructan, *Limisolactobacillus fermentum* (von Weymarn et al. 2003; Martínez‐Miranda et al. 2022), produced less than 3 g/L mannitol from YMRS. However, the bacteria produced at least 10 g/L when 1% fructose was used as a substrate in FMRS after 20 h (Figure S1), suggesting that the floral FLAB isolates were better suited to convert the untreated FOS to mannitol. We therefore further investigated the production of mannitol from YRP using the FLAB. Interestingly, these fermentations revealed that the best mannitol producers from YRP did not correlate with the best mannitol production from fructose as previously reported.

**Table 1:** List of bacteria used in this study.

Species	Strain	Isolated from	Source
*Leuconostoc mesenteroides*	DSM 20343^T^	Fermenting olives	DSMZ
*Leuconostoc mesenteroides*	DPC 7246*	Cotoneaster flowers	DPC Culture Collection, Teagasc, Fermoy, Ireland
*Leuconostoc mesenteroides*	DPC 7255*	Dandelion flowers	DPC Culture Collection, Teagasc, Fermoy, Ireland
*Leuconostoc mesenteroides*	DPC 7261*	Cotoneaster flowers	DPC Culture Collection, Teagasc, Fermoy, Ireland
*Leuconostoc mesenteroides*	DPC 7263*	Laurel leaves	DPC Culture Collection, Teagasc, Fermoy, Ireland
*Leuconostoc mesenteroides*	DPC 7267*	White clover flowers	DPC Culture Collection, Teagasc, Fermoy, Ireland
*Fructobacillus fructosus*	DSM202349^T^	Flowers	DSMZ
*Fructobacillus fructosus*	DPC 7235*	White clover flowers	DPC Culture Collection, Teagasc, Fermoy, Ireland
*Fructobacillus fructosus*	DPC 7237*	White clover flowers	DPC Culture Collection, Teagasc, Fermoy, Ireland
*Limisolactobacillus fermentum*	DSM 20391	Unknown	DSMZ

* First described in (Behare et al. 2020a).

All the selected FLAB were previously identified as heterofermentative LAB (Behare et al. 2020a), which are best suited for mannitol production from fructose as the latter sugar is unlikely to be used for growth. Accordingly, sugars in the YRP were found to be the main substrate for mannitol production. Growth of the same FLAB in MRS without fructose or YRP significantly decreased mannitol production. In comparison to inulin, YRP produced significantly more mannitol for the same fermentation time, suggesting that the FOS was more bioavailable as a substrate for mannitol than inulin (Figure S2).

Following these experiments, we focused on two FLAB: *F. fructosus* DPC 7237, isolated from white clover flowers and *L. mesenteroides* DPC 7246, isolated from cotoneaster flowers. While all *L. mesenteroides* strains screened produced approximately the same amount of mannitol, *L. mesenteroides* DPC 7246 exhibited more robust growth under the conditions of interest and was a less stringent microaerophile in terms of oxygen tolerance, leading us to select this strain. However, *F. fructosus* strains flocculated strongly in a manner independent of agitation. While this trait could simplify downstream processing in future studies, at this stage it would also complicate cell density measurement by OD and consistent inoculations from precultures. As a result we focused on *L. mesenteroides* DPC 7246 for scaling up mannitol production from YRP while still investigating optimal culture conditions for *F. fructosus*.

### 3.3 Effects of Fermentation Time on Mannitol Production

**Figure 2:**
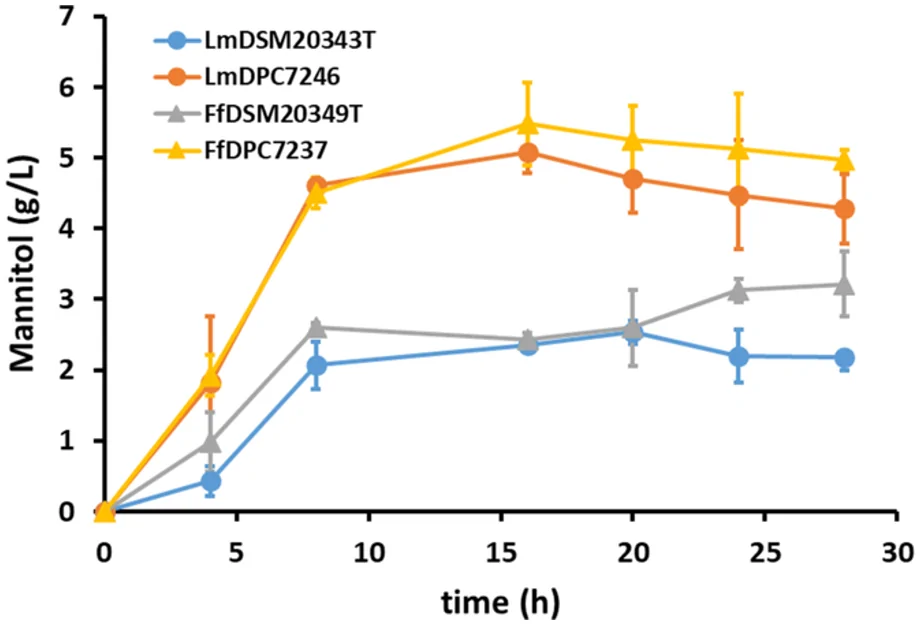
Time course of mannitol production from 1% YMRS. The fructophilic lactic acid bacteria selected from the initial screening produced nearly twice as much mannitol as their respective type species (identified by “T”). 15 mL cultures were sampled in triplicate at the indicated time points and mannitol quantified by HPLC. *L. mesenteroides* (Lm) strains are plotted as filled circles, and *F. fructosus* (Ff) strains as filled triangles. Data are plotted as mean ± s.d. of three replicates as error bars.

Previous work using the same strains reported an optimal mannitol production at 20 h with 1% fructose as a substrate (Behare et al. 2020a). As we intended to evaluate a more complex carbohydrate as a substrate that might require longer processing before conversion to mannitol, we measured the mannitol production of the most promising FLAB strains from 1% YMRS over time for 28 h to identify the optimal production time. The time‐course experiments revealed that to be between 16 and 20 h depending on the strain used (Figure 2 **)**, with peak titers of 5.1 ± 0.48 g/L for *L. mesenteroides* DPC 7246 and 5.5 ± 0.58 g/L for *F. fructosus* DPC 7237 but a uniform decrease after 24 h fermentation was observed. Although the enzymatic conversion of fructose to mannitol is reversible, the absence of detectable fructose alongside decreasing mannitol levels may suggest that mannitol was reconverted to fructose to support central carbon metabolism or cofactor regeneration.

### 3.4 Effects of Temperature and Agitation on Mannitol Production

**Figure 3:**
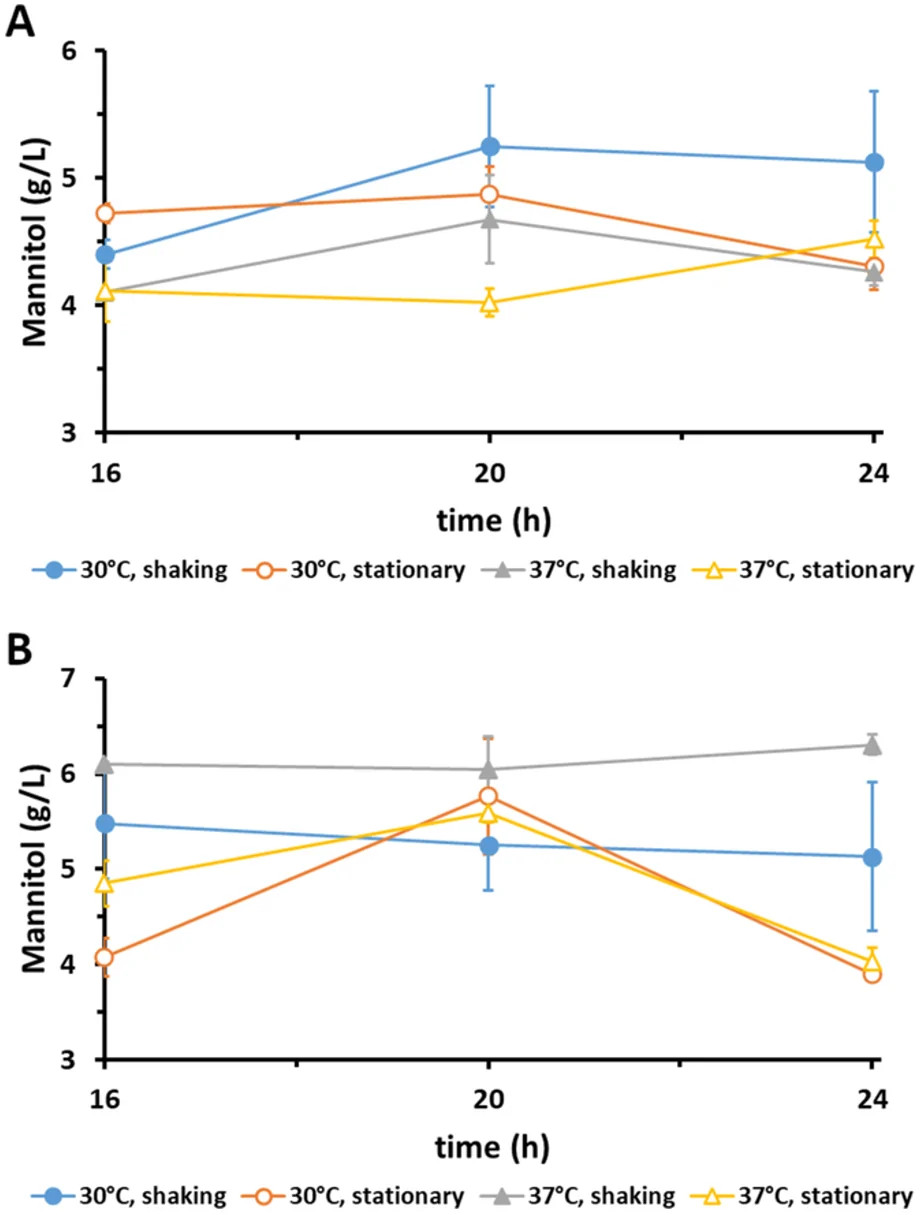
Effects of temperature and agitation on mannitol production. *L. mesenteroides* DPC 7246 (a) and *F. fructosus* DPC 7237 (b) were grown in 1% YMRS at 30 or 37°C and with or without shaking. Samples were taken for mannitol estimation at time points before and after the optimal culture time for production at 30°C. Data are plotted as mean ± s.d. of three replicates as error bars.

The experiments to date had evaluated mannitol production aerobically (cultured with agitation) at 30°C. However, as other FLABs from the same sources had been identified to be microaerophilic, fermentations were also conducted with and without agitation to determine if the lower aeration brought on by the latter condition improved mannitol production (Figure 3). In general, changing shaking conditions or increasing fermentation temperature did not significantly improve mannitol production. While there were no decreases in mannitol production greater than 25% across all conditions for *L. mesenteroides* DPC 7246, fermentations without agitation had lower maximum titers by 8% at 30°C and 14% at 37°C. In the case of *F. fructosus* DPC 7237, production without agitation peaked much more sharply than cultures with agitation. Furthermore, cultures at 37°C yielded the maximum mannitol at 20 h as at 30°C. As there was no clear advantage to changing temperature or agitation to improve mannitol production based on these results, further experiments were performed at 30°C with mild agitation.

### 3.5 Effects of Yacon Root Powder Loading on Mannitol Production

**Figure 4:**
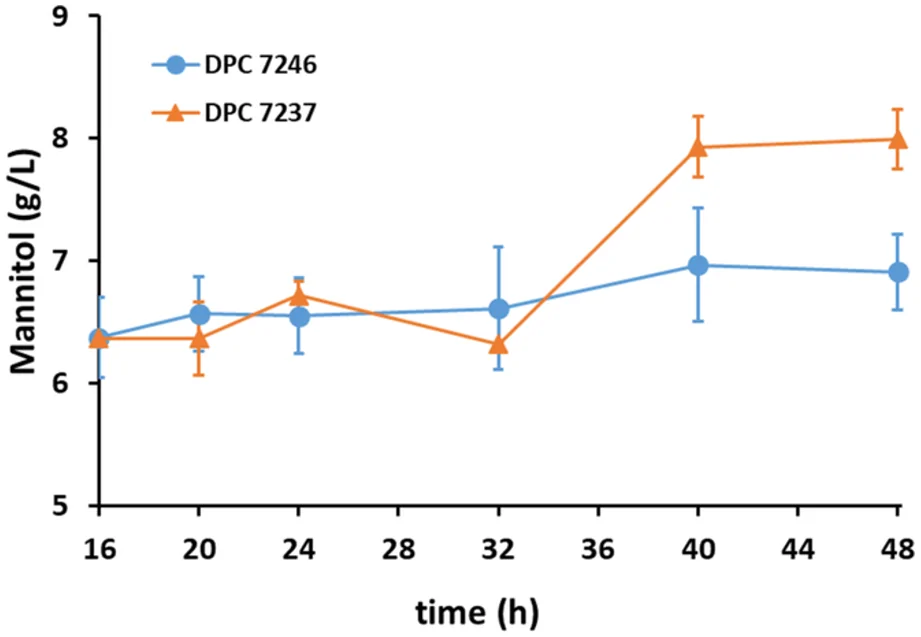
Production of mannitol on 2% YMRS. *F. fructosus* DPC 7237 and *L. mesenteroides* DPC 7246 fermented MRS with 2% YRP (2% YMRS) for 48 h at 30°C with 150 rpm shaking. Data are plotted as mean ± s.d of three replicates as error bars.

On increasing YRP in the medium to 2% (2% YMRS), peak mannitol titers for DPC 7246 only increased by about 33% (Figure 4). This was accompanied by detectable ethanol production. Examining time‐dependent production, we found that titers peaked at approximately 32 h, staying constant or slightly decreasing up to 48 h. While ethanol is an expected by‐product of heterofermentative LAB (Wisselink et al. 2002), it was not previously measured when fermenting 1% YMRS by any FLAB. It is possible that some of the fructose from the YRP (either free or hydrolyzed from FOS) overflows from mannitol production and is funnelled into the pentose phosphate pathway (Årsköld et al. 2008), ultimately ending up as ethanol. It may also be that the increased YRP metabolism results in a higher amount of NADH production, causing the bacterium to use ethanol production to regenerate NAD^+^. This was coupled to a decrease in acetic acid production and an increase in lactic acid production compared to growth 1% YRP (Figure S3a). While both lactic and acetic acid were observed when DPC 7246 fermented 1% or 2% YMRS, concentrations of the former increased and the latter decreased at 2% YMRS. The net result is a decrease in the yield of mannitol from YRP at peak titer, decreasing from 0.92 g mannitol/g FOS on 1% YRP to 0.635 g mannitol/g FOS on 2% YRP.

### 3.6 Scale‐Up of Production of Mannitol

**Figure 5:**
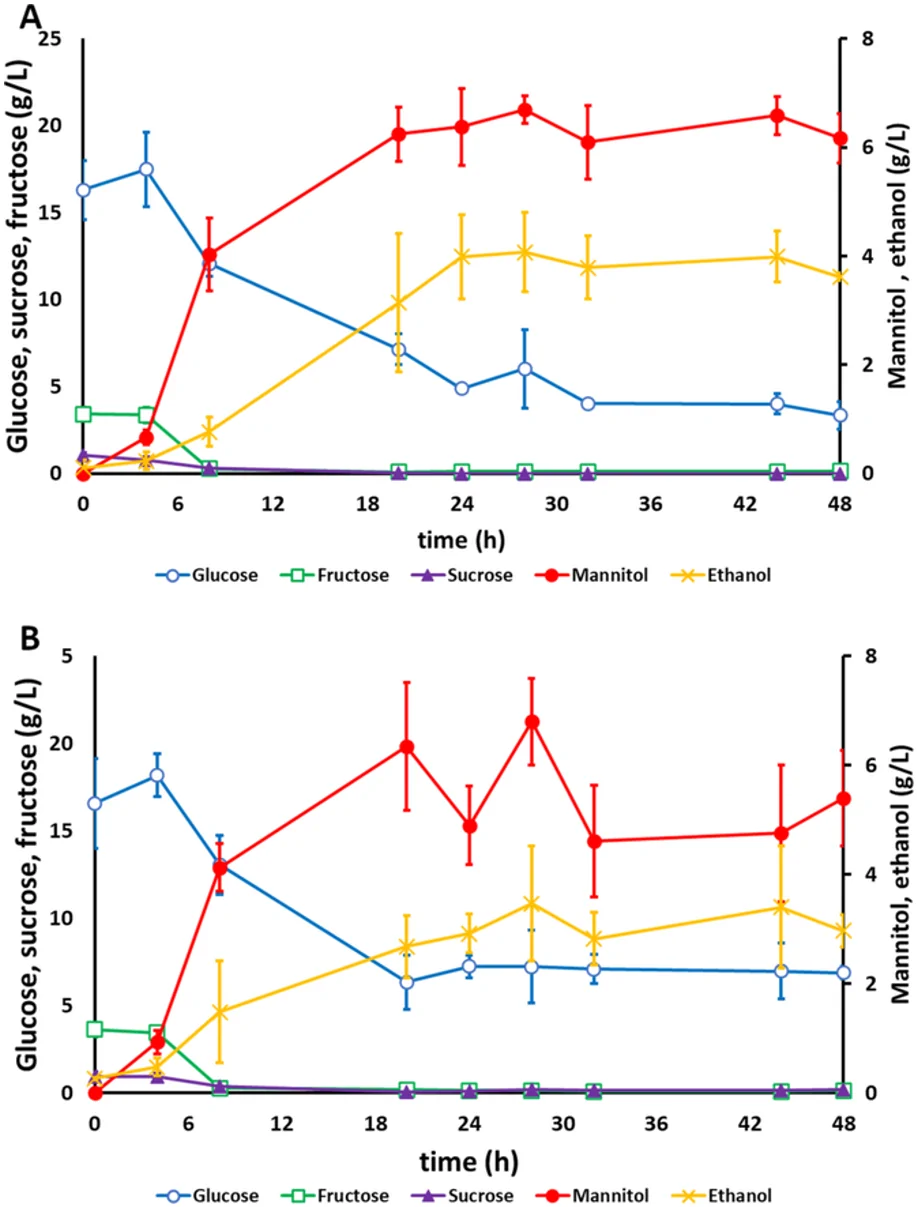
Batch fermentation of 2% YMRS by *L. mesenteroides* DPC 7246 in benchtop fermenters Fermentations were carried out at (a) 30°C and (b) 37°C in 200 mL volumes without aeration or pH control. Consumed sugars are plotted on the left *y*‐axis and alcohols produced on the right *y*‐axis, and are plotted as mean ± s.d. of three independent experiments as error bars.

**Figure 6:**
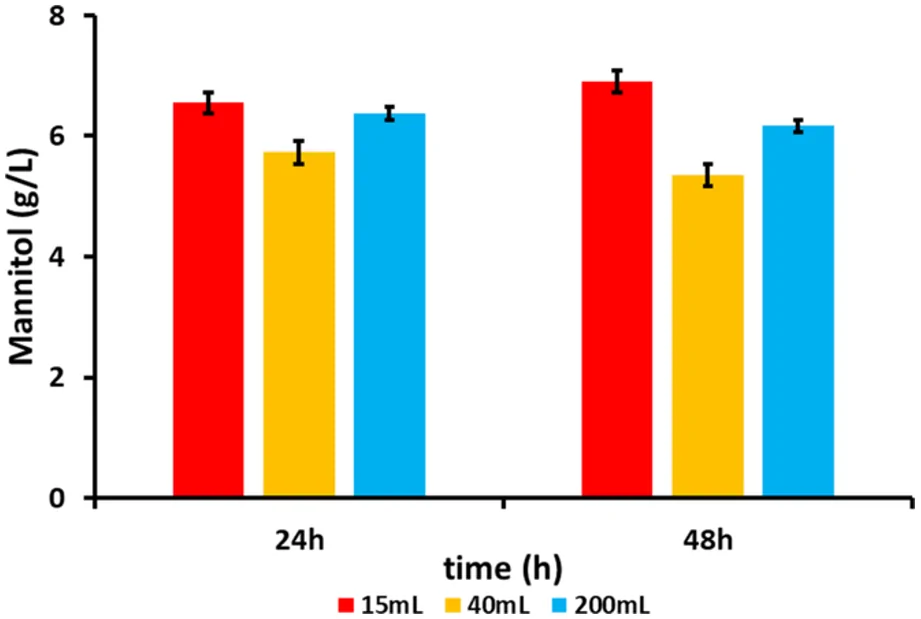
Production of mannitol by *L. mesenteroides* DPC 7246 at different scales. Differences in mannitol production on 2% YRP were more pronounced at 48 h. Data are plotted as the mean ± s.d. of three replicates as error bars.

Experiments to date had been conducted with 15 mL fermentation volumes. Using benchtop fermenters, we evaluated 200 mL cultures in YMRS for the production of mannitol at 30°C and 37°C. Besides the tenfold increase in volume in a new vessel geometry, the scale‐up also allowed us to better control the heating and mixing regimes, as well as altered headspace in the culture vessel. As previously reported (Cao et al. 2018), the cultures were agitated but not aerated and pH was not controlled. At the end of a fermentation, the pH of the medium was typically between 4.1 and 4.3. The results are reported in Figure 5. Under these conditions, mannitol production peaked at 6.7 g/L, roughly the same as at lower culture volumes. However, the fermentation reached its maximum titer earlier than at smaller scale (24–28 h vs. 32 h at 15 mL) before levelling off. A similar trend was seen in ethanol levels. While mannitol production decreased when scaling up from 15 to 200 mL cultures (Figure 6), we also investigated mannitol production at 40 mL post hoc to examine the scaling of production in greater detail. While mannitol titers did not significantly change when scaling from 15 mL (6.55 g/L at 24 h) to 200 mL (6.38 g/L at 24 h), both culture volumes produced higher titers than in shake flask cultures at 40 mL (5.73 g/L at 24 h). It is likely that the extra aeration at this volume affected the redox balance and reduced the need for NAD^+^ regeneration. Biomass did not significantly change as temperature was increased (Figure S4), with cultures reaching up to 2 × 10^8^ cfu/mL at peak mannitol production.

Mannitol titers in a 37°C fermentation increased up to 20–24 h after which it fluctuated, which may be due to cycling of the mannitol dehydrogenase reaction at this elevated temperature. In contrast, ethanol titers at 37°C were lower by about 20% when compared to 30°C but glucose consumption was slower: approximately 6.9 g/L of the glucose remained after 48 h at 37°C as opposed to 3.3 g/L at 30°C. Finally, the scaling up of fermentation did not increase the production of lactic acid at either temperature (Figure S3b). Accounting for the percentage of FOS present in 2% YRP, we calculate our peak mannitol yields for DPC 7246 to be 0.61–0.62 g mannitol/g FOS at both temperatures. The yield from 15 mL cultures was 0.63 g/g, suggesting that scale‐up did not significantly affect production. Encouragingly, these yields are within the range reported in other studies on saccharified fructan substrates: 0.58 g/g from inulin (Saha 2006b) 0.69 g/g from chicory hydrolysate or cashew juice (Fontes et al. 2009; Zhang et al. 2017), 0.68 g/g from Jerusalem artichoke (Cao et al. 2018). However, fermentations using hydrolyzed fruit juices have achieved higher yields in excess of 0.7 g mannitol/g fructose (Carvalheiro et al. 2011). The mannitol titers we report on YRP are also comparable or superior to those reported for more complex, unsaccharified or unhydrolysed feedstocks in the literature (Table 2): fermentation of Jerusalem artichoke juice by *L. paracasei* subsp. *paracasei* and *L. casei* produced up to 3. g/L mannitol after a 20 h fermentation (Zalán et al. 2011), compared to 4–5 g/L from our screened *L. mesenteroides* and *F. fructosus* strains. It is likely that the smaller size of yacon FOS allows for its efficient hydrolysis and simultaneous saccharification and fermentation by *L. mesenteroides*. Among complex or second‐generation feedstocks, another study used grape musts as a substrate to yield over 60 g/L mannitol from 100 g/L must, though the fermentation ran for over 168 h (Hijosa‐Valsero et al. 2021). Higher FOS loading could achieve similar titers in potentially shorter times, given the current yields achievable. We also acknowledge that some form of hydrolytic pre‐treatment may be required to optimize yields, as has been seen when fruit or vegetable pulp was used as a substrate for mannitol (Molina et al. 2023). Such hydrolysis may be essential if we intend to valorize yacon residue after syrup extraction and powder formulation.

**Table 2:** Reported mannitol production and yields from complex plant‐based substrates.

Host	Substrate	Fermentation temperature/time	Maximum mannitol		Reference
			Yield	Titer	
*Lactobacillus intermedius*	30% hydrolyzed inulin/fructose	37°C, 96 h	0.58 g/g	227.9 g/L	Saha (2006a)
*Leuconostoc mesenteroides*	Cashew apple juice	30°C, 10 h	0.62 g/g	18 g/L	Fontes et al. (2009)
*Leuconostoc fructosum*	33% carob syrup hydrolysate	30°C, 24 h	1.06 g/g	43.7 g/L	Carvalheiro et al. (2011)
*Leuconostoc mesenteroides* subsp. *cremoris*			0.83 g/g	~32 g/L	
*Lactobacillus casei*	10% Jerusalem artichoke juice	37°C, 72 h	n.a.	3.6 g/L	Zalán et al. (2011)
*Leuconostoc pseudomesenteroides*	Inulin hydrolysate from chicory	30°C, 60 h	0.69 g/g	64.6 g/L	Zhang et al. (2017)
*Lactobacillus intermedius*	Grape musts and wine lees	30°C,144 h	0.66* g/g	65.6 g/L	Hijosa‐Valsero et al. (2021)
*Leuconostoc mesenteroides*	Yacon root powder	30°C, 48 h	0.61 g/g	6.7 g/L	This study

* Calculated from sugar content, conversion date and titers provided in the paper.

The results reported here serve as a proof of concept for the production of mannitol from yacon. To make production more economically viable and competitive, future work would focus on the optimization of fermentation to yield higher titers. These would necessarily require higher YRP concentrations in fermentations, tied to further investigations on *L. mesenteroides* strains or even *F. fructosus* with higher fructose tolerance than what was reported with DPC 7246. Based on initial screening, substrate utilization, robust growth and flocculation characteristics, the strain we selected to demonstrate mannitol production from yacon was unexpectedly not the strongest mannitol producer the literature previously identified. However, as *L. mesenteroides* produced ethanol as a side‐product of its metabolism with 2% YMRS, future work would also need to evaluate alternate substrate feeding strategies to ensure that the higher amounts of substrate are efficiently converted to mannitol. Initial fermentations revealed a more efficient production of mannitol (0.92 g/g vs. 0.61 g/g) at lower YRP loading as well as less ethanol and lactic acid being produced over mannitol; using a fed‐batch fermentation may allow a higher yield of mannitol without a loss of productivity. Higher substrate loading, facilitated by YRP's high solubility, and scale‐up may also require different medium formulations to bring down ingredient costs and avoid the problem of precipitate formation, especially as MRS is a relatively complex culture medium. Previous work on medium optimization suggests tryptone and corn steep liquor may serve as promising alternate nitrogen sources for lactic acid bacteria (Saha 2006a); such ingredients will be investigated in future studies involving YRP and FLABs.

## 4 Conclusion

In this study, we demonstrated for the first time that yacon fructooligosaccharides were a viable substrate for efficient mannitol production using fructophilic lactic acid bacteria at yields of 0.61–0.63 g mannitol/g FOS, comparable to those reported for other complex substrates (Table 2). In doing so, we also added economic value to the yacon tuber beyond its value as a staple and health food. As yacon remains a niche food in developed countries and has recently shown promise as a crop in Ireland (Beotanics 2019), its use in the sustainable production of mannitol, which has multiple applications in the food and pharma industry (Martínez‐Miranda et al. 2022) is unlikely to affect availability or supply in areas where it is a dietary staple. We have used yacon root powder, containing a purely natural, plant‐based fructooligosaccharide as a substrate for this enzyme *in vivo* and successfully produced mannitol from a *Leuconostoc mesenteroides* strain from 2% YRP at multiple scales with no significant decrease in a maximum titer of 6.7 g/L mannitol on scaling up. Our strains utilize intact fructooligosaccharide from yacon as a substrate whereas most past studies on mannitol production used hydrolyzed fructose‐rich substrates, mixtures of reducing sugars or natural fruit syrups or juices rich in reducing sugars. In summary, our results provide an important first step in exploiting a natural substrate for the bacterial production of mannitol and respectively demonstrate the viability of yacon fructooligosaccharides and fructophilic lactic acid bacteria for this task.

## Author Contributions

**Arun S. Rajkumar:** investigation, methodology, validation, conceptualization; formal analysis, writing – review and editing, writing – original draft, visualization. **John Leech:** writing – review and editing, investigation;, resources, methodology. **Olivia McAuliffe:** conceptualization, methodology, investigation, supervision, resources, project administration, writing – original draft, writing – review and editing, funding acquisition.

## Ethics Statement

The authors have nothing to report.

## Conflicts of Interest

The authors declare no conflicts of interest.

## Supporting information


Supporting File

## Data Availability

The data that support the findings of this study are available on request from the corresponding author within reasonable limits. Microbial Biotechnology (MBT2).
