# Supplementary material for: Single‐Step Production of Mannitol From Yacon Fructo‐Oligosaccharides Using Fructophilic Lactic Acid Bacteria
Source: Microbiologyopen. 2026 Sep 22;15(5):e70387. doi: 10.1002/mbo3.70387 (PMC13598372; doi:10.1002/mbo3.70387)
Supplement: Supplementary file 1 [file MBO3-15-e70387-s001.docx]

**SUPPORTING FIGURES**

**Figure S1. Production of mannitol from fructose by fructophilic lactic acid bacteria.** The strains were cultured in MRS broth containing 1% fructose (1% FMRS) and mannitol measured in the medium after 20h culture as described in the Methods. Data are plotted as the mean ± s.d. of duplicates and the bars are coloured by species as in Figure 1.

**Figure S2. Production of mannitol from diverse carbon sources.**  The selected FLAB strains were grown in an MRS base with different amounts of glucose (glu), fructose (fru), yacon root powder (YRP) or inulin. The first medium corresponds to standard MRS. The data are plotted as the mean ± s.d. of duplicates at 24h, as the cultures grown on YRP without glucose grew more slowly than the others.

**Figure S3. Secondary metabolite production by *L. mesenteroides* DPC 7246.** Increasing either (A) YRP concentration and (B) culture volume increases the production of ethanol and lactic acid.

**Figure S4**. **Growth of *Leuconostoc mesenteroides* DPC 7246 in benchtop fermenters.** Cultures were set up as described in Materials and Methods at 30°C (blue filled circles) and 37°C (orange filled circles) and OD measured at the indicated time points. Data are plotted as the mean ± s.e.m. of duplicates.
